# Racial and Ethnic Inequalities for Nonfatal Legal Intervention Injuries Treated in US Emergency Departments

**DOI:** 10.1001/jamanetworkopen.2025.41166

**Published:** 2025-10-30

**Authors:** Mina Kim, Phillip Atiba Solomon, Justin M. Feldman

**Affiliations:** 1 Center for Policing Equity, West Hollywood, California; 2 Department of African American Studies, Yale University, New Haven, Connecticut; 3 Department of Psychology, Yale University, New Haven, Connecticut; 4 François-Xavier Bagnoud Center for Health and Human Rights, Harvard University, Cambridge, Massachusetts

## Abstract

This cross-sectional study explores racial and ethnic disparities in injuries caused by law enforcement and treated in US emergency departments (EDs) from 2004 to 2021.

## Introduction

In the US, injuries caused by law enforcement are a public health concern and driver of racial health inequities.^1,2,3,4^ While fatalities have attracted considerable public attention, nonfatal injuries inflicted by police are far more prevalent. Analyzing nonfatal injury trends can therefore help to answer critical questions about how populations experience policing, including whether the major advocacy efforts related to racial inequity in policing occurring from 2014 to 2021^5^ coincided with changes to rates of legal intervention injury overall or by racial and ethnic group.

## Methods

This repeated cross-sectional study analyzed publicly accessible, deidentified data from the National Electronic Injury Surveillance System—All Injuries Program (NEISS-AIP), a nationally representative sample of US hospital emergency departments (EDs), for the period 2004 to 2021. Local ethics review and informed consent were not required in accordance with the Common Rule. This study followed the Strengthening the Reporting of Observational Studies in Epidemiology (STROBE) reporting guideline for cross-sectional studies.

In NEISS-AIP, *legal intervention* includes an injury or poisoning caused by on-duty police or other legal authorities, including private security guards. NEISS-AIP offers an advantage over administrative claims data, which underreport substantial shares of legal intervention injuries.^6^ We used NEISS-AIP’s predefined race and ethnicity categories, which were derived from patient medical records (eAppendix in Supplement 1).

All analyses were performed between September 2024 and July 2025 using R software version 4.2 (R Project for Statistical Computing) with the survey and mgcv packages. We fit quasi-Poisson models for injury rates, treating year as a spline (for visualization) or as linear (to assess trends quantitatively), using parametric bootstrapping to construct CIs. We deemed any 95% CI for the linearized trend line that included the null value to be inconclusive as to its directionality, but we still interpreted the confidence limit as the bounds within which the trend was expect to fall. Additional methodological details are available in the eAppendix in Supplement 1.

## Results

Between 2004 and 2021, a total of 1 500 577 ED visits (95% CI, 1 073 632-1 927 522; 85% [95% CI, 60% to 100%] men; mean [SD] age, 33 [12] years) in the US were for legal intervention injuries. Among patients with race and ethnicity data, 42.3% (95% CI, 21.2% to 63.4%) were African American or Black, 13.9% (95% CI, 5.4% to 22.3%) were Hispanic or Latinx, and 41.1% (95% CI, 30.7% to 51.5%) were White. Most patients were treated and released, with only 4.3% (95% CI, 2.5% to 6.1%) requiring hospitalization (Table).

**Table.** Legal Intervention Injuries in Hospital Emergency Departments, 2004-2021

Characteristic	Legal intervention injuries	
	No. (95% CI)	% (95% CI)^a^
Sex^b^		
Men	1 270 350 (896 648-1 644 051)	84.7 (59.8-100)^c^
Women	229 863 (173 663-286 062)	15.3 (11.6-19.1)
Race and ethnicity^d^		
African American or Black	494 432 (247 922-740 942)	42.3 (21.2-63.4)
Hispanic or Latinx	161 917 (62 633-261 201)	13.9 (5.4-22.3)
White	480 110 (358 368-601 852)	41.1 (30.7-51.5)
Other races	32 777 (12 232-53 321)	2.8 (1.0-4.6)
Age, y^e^		
≤14	26 349 (15 453-37 246)	1.8 (1.0-2.5)
15-24	424 842 (302 945-546 739)	28.3 (20.2-36.5)
25-34	469 617 (334 934-604 300)	31.3 (22.3-40.3)
35-44	312 805 (216 562-409 048)	20.9 (14.4-27.3)
45-54	182 773 (130 607-234 939)	12.2 (8.7-15.7)
≥55	83 274 (61 688-104 859)	5.6 (4.1-7)
Disposition of patient^f^		
Treated and released	1 335 886 (972 719-1 699 054)	89.0 (64.8-113.2)
Transferred and released	27 617 (19 403-35 830)	1.8 (1.3-2.4)
Hospitalized	64 505 (38 009-91 002)	4.3 (2.5-6.1)
Observation	46 236 (−34 468-126 939)	3.1 (0-8.5)^c^
Left against medical advice/without being seen	26 245 (11 195-41 294)	1.8 (0.8-2.8)
Immediate cause of injury^g^		
Cut/pierce	141 651 (41 847-241 455)	9.5 (2.8-16.2)
Dog bite	70 931 (33 307-108 555)	4.8 (2.2-7.3)
Fall	214 262 (152 156-276 368)	14.4 (10.2-18.6)
Gunshot	19 285 (3701-34 869)	1.3 (0.3-2.3)
Struck by/against	817 528 (591 204-1 043 851)	55.0 (39.8-70.2)
Other	223 345 (167 256-279 434)	15.0 (11.3-18.8)
Primary diagnosis		
Chemical burn	15 801 (9682-21 920)	1.1 (0.7-1.5)
Concussion	14 610 (11 002-18 219)	1.0 (0.7-1.2)
Contusion/abrasion	511 513 (353 623-669 403)	34.1 (23.6-44.6)
Foreign body	56 687 (20 660-92 713)	3.8 (1.4-6.2)
Fracture	111 011 (85 987-136 034)	7.4 (5.7-9.1)
Internal injury	131 674 (89 727-173 621)	8.8 (6.0-11.6)
Laceration	184 384 (132 325-236 444)	12.3 (8.8-15.8)
Poisoning	27 022 (20 402-33 642)	1.8 (1.4-2.2)
Puncture	98 591 (1629-195 552)	6.6 (0.1-13)
Strain/sprain	181 517 (140 171-222 862)	12.1 (9.3-14.9)
Other	167 767 (123 903-211 630)	11.2 (8.3-14.1)
Total	1 500 577 (1 073 632-1 927 522)	NA

Abbreviation: NA, not applicable.

^a^ The denominator only includes legal intervention injuries with non-missing data for the respective variable.

^b^ A total of 0.02% of individuals had unknown or nonbinary sex. The nonbinary category was only available in the 2021 data.

^c^ 95% CIs for percentage that extend above 100% or below 0% have been truncated.

^d^ African American or Black includes Black Hispanic or Latinx individuals; Hispanic or Latinx excludes Black Hispanic or Latinx individuals. White excludes White Hispanic or Latinx. Other races include American Indian or Alaska Native, Asian, Pacific Islander, and more than 1 race. Data were missing for 22.08% of individuals.

^e^ Data were missing for 0.06% of individuals.

^f^ Data were missing for 0.01% of individuals.

^g^ Data were missing for 0.90% of individuals.

Legal intervention injury rates for the US population as a whole remained relatively stable over the study period (Figure), with the 2021 rate at 92% (95% CI, 71% to 119%) the level of the 2004 rate. Over the study period, mean injury rates for African American or Black people were 5.3 (95% CI, 4.6 to 6.2) times those of White people. Rates for Hispanic or Latinx people were 1.5 (95% CI, 1.2 to 1.7) times those of White people. While the point estimates for Black:White RR decreased by 6%, from 5.48 in 2004 to 5.13 in 2021, uncertainty was high, with the 95% CI ranging from a 42% reduction to 51% increase. For the Latinx population, the RR decreased from 1.94 in 2004 to 1.06 in 2021, corresponding to a 45% (95% CI, 12% to 66%) decrease.

**Figure.**
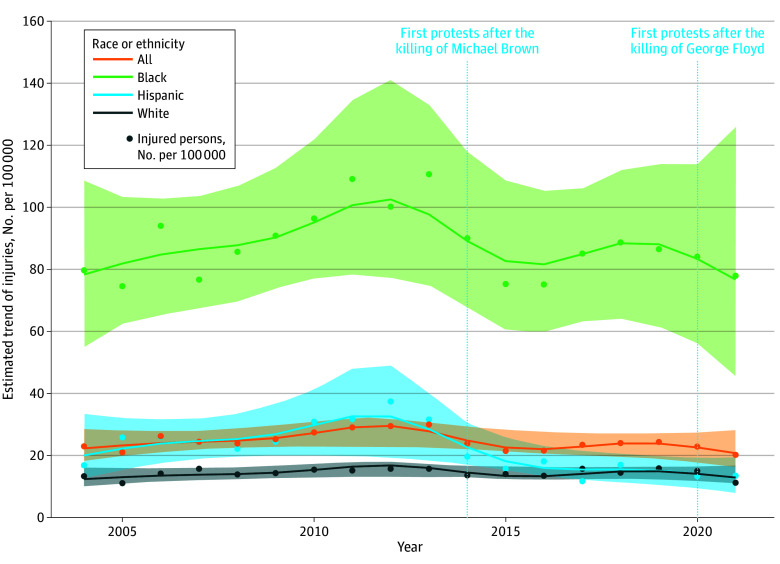
Yearly Estimated Legal Intervention Injury Trend by Race and Ethnicity

## Discussion

Despite expectations that policy responses to major protests against racialized police violence would lead to substantial change in the rate of legal intervention injuries, the findings of this cross-sectional study suggest that any shifts for the overall population were relatively modest, ranging from a decrease by approximately one-third to an increase of one-fifth. Our findings of a decreasing RR for Hispanic or Latinx individuals compared with White individuals should be interpreted cautiously, as misclassification of Hispanic or Latinx patients in US medical data is high and may vary as EDs with different classification practices enter and exit the NEISS-AIP sample. While we cannot draw conclusions about the direction of change to the RR for African American or Black individuals, it is clear that it remained persistently high over the entire study period, with point estimates always exceeding 5.

## Notes

Supplement 1. **eAppendix.** Supplementary Methods

Supplement 2.
Data Sharing Statement
