# Supplementary material for: Racial and Ethnic Inequalities for Nonfatal Legal Intervention Injuries Treated in US Emergency Departments
Source: JAMA Netw Open. 2025 Oct 30;8(10):e2541166. doi: 10.1001/jamanetworkopen.2025.41166 (PMC12576483; doi:10.1001/jamanetworkopen.2025.41166)
Supplement: Supplement 2. — Data Sharing Statement [file jamanetwopen-e2541166-s002.pdf]

# Data Sharing Statement

Kim. Racial and Ethnic Inequalities for Nonfatal Legal Intervention Injuries Treated in US Emergency Departments. *JAMA Netw Open*. Published October 30, 2025. doi:10.1001/jamanetworkopen.2025.41166

**Data**
**Data available:** Yes
**Data types:** Data (not involving human participants)
**How to access data:** https://github.com/mak791/NEISS
**When available:** With publication

**Supporting Documents**
**Document types:** Statistical/analytic code
**How to access documents:** https://github.com/mak791/NEISS
**When available:** With publication

**Additional Information**
**Who can access the data:** Anyone requesting the data
**Types of analyses:** Any purpose
**Mechanisms of data availability:** All data and code are open-access and available at the URL provided
